# A Portable Wireless Device for Cyclic Alternating Pattern Estimation from an EEG Monopolar Derivation

**DOI:** 10.3390/e21121203

**Published:** 2019-12-07

**Authors:** Fábio Mendonça, Sheikh Shanawaz Mostafa, Fernando Morgado-Dias, Antonio G. Ravelo-García

**Affiliations:** 1 Instituto Superior Técnico, University of Lisbon, 1049-001 Lisbon, Portugal; sheikh.mostafa@tecnico.ulisboa.pt; 2 Madeira Interactive Technologies Institute (ITI/Larsys/M-ITI), 9020-105 Funchal, Portugal; morgado@uma.pt; 3 Faculty of Exact Sciences and Engineering, University of Madeira, 9000-082 Funchal, Portugal; 4 Institute for Technological Development and Innovation in Communications, Universidad de Las Palmas de Gran Canaria, 35001 Las Palmas de Gran Canaria, Spain; antonio.ravelo@ulpgc.es

**Keywords:** sleep quality, EEG, CAP, GRU, LSTM, 1D-CNN

## Abstract

Quality of sleep can be assessed by analyzing the cyclic alternating pattern, a long-lasting periodic activity that is composed of two alternate electroencephalogram patterns, which is considered to be a marker of sleep instability. Experts usually score this pattern through a visual examination of each one-second epoch of an electroencephalogram signal, a repetitive and time-consuming task that is prone to errors. To address these issues, a home monitoring device was developed for automatic scoring of the cyclic alternating pattern by analyzing the signal from one electroencephalogram derivation. Three classifiers, specifically, two recurrent networks (long short-term memory and gated recurrent unit) and one one-dimension convolutional neural network, were developed and tested to determine which was more suitable for the cyclic alternating pattern phase’s classification. It was verified that the network based on the long short-term memory attained the best results with an average accuracy, sensitivity, specificity and area under the receiver operating characteristic curve of, respectively, 76%, 75%, 77% and 0.752. The classified epochs were then fed to a finite state machine to determine the cyclic alternating pattern cycles and the performance metrics were 76%, 71%, 84% and 0.778, respectively. The performance achieved is in the higher bound of the experts’ expected agreement range and considerably higher than the inter-scorer agreement of multiple experts, implying the usability of the device developed for clinical analysis.

## 1. Introduction

Sleep is a complex, dynamic process inherent to each individual that is part of the circadian rhythm and is commonly characterized by sequences of stages that are related to autonomous nervous system functions [1]. It is frequently evaluated by sleep related variables that are attained from examining the signals recorded through polysomnography (PSG), considered to be the gold standard for sleep analysis [2].

PSG is a technique that comprises the employment of multiple sensors for accurate sleep analysis [3]. The electroencephalogram (EEG) signals are the references to define the sleep structure that are conventionally divided into the macro and the microstructure. The macrostructure is characterized by repeated variations, scored in 30 s epochs, of rapid eye movement (REM) and non-REM (NREM) [2].

**Figure 1:**
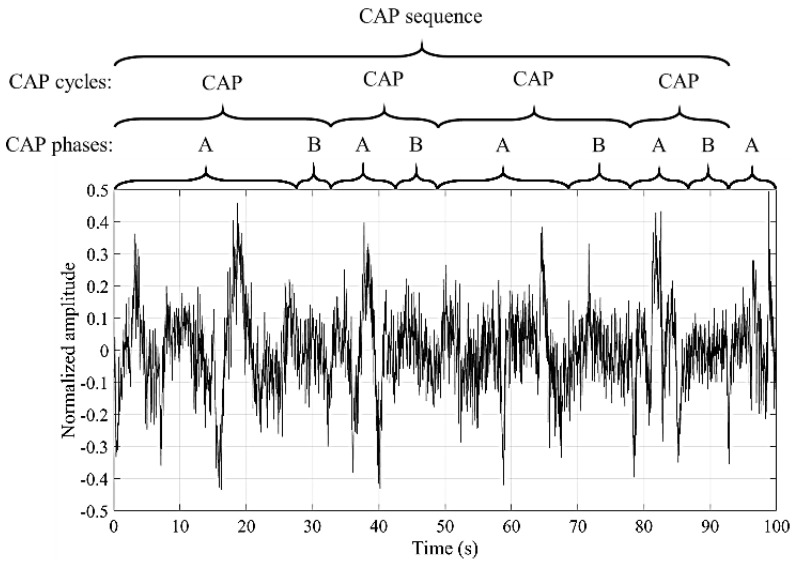
Example of the EEG signal classification into the cyclic alternating pattern (CAP) phases, cycles and sequence.

The microstructure is characterized by the transient and phasic events in neural electrical activity, scored in 1 second epochs [4]. A way of analyzing this structure, in the NREM sleep stages, was defined by Terzano et al. [5] through the cyclic alternating pattern (CAP). This pattern is composed by cycles of activations (named A phases) that are followed by quiescent phases (named B phase) [6]. A CAP cycle is composed of an A phase followed by a B phase and the duration of each phase ranges between two and sixty seconds. A succession of two or more CAP cycles produces a CAP sequence [7]. An explanatory example regarding the classification of an EEG signal into the CAP phases, cycles and sequence is presented in Figure 1. The A phases can be subdivided into three subtypes (A1, A2 and A3) [5] that can be examined for a deeper analysis of the sleep process [8,9,10].

The quality of sleep can be estimated by PSG-based sleep quality measures [11]. CAP is considered a sleep instability metric since a disturbance in sleep generates distinguishing alterations in neural electrical activity that, usually, produce an A phase [12]. Therefore, the assessment of the CAP cycles can be employed to estimate the sleep quality. In healthy adults, a cycle lasts, on average, 26.9 ± 4.1 seconds and a usual CAP sequence is composed of 5.6 CAP cycles [7]. The A phase subtypes were not considered for this work since the goal was to estimate the CAP phases and cycles as metrics for sleep quality analysis.

The major concern regarding CAP examination is the fact that it, generally, is scored through visual examination of the EEG signal. This process is considered to be a time-consuming and tedious task that is prone to error due to the concentration loss of the physician [13]. Consequently, the mutual agreement among two physicians, examining the same EEG signals, ranges from 69% to 77.5% [14]. The agreement gets closer to the lower bound as more physicians are considered in the agreement analysis, as reported by Largo et al. [15], where the global average of the pairwise inter-scorer agreement of seven experts was 69.9%. Therefore, an automatic scorer algorithm implemented in a home monitoring device (HMD) is desired to address this issue. It could possibly allow the detection and treatment of sleep quality deficits in a large group of the world population that does not have access to a PSG.

Two main approaches have been proposed which are the state of the art regarding the CAP cycles’ estimation [16]. The first method is comprised of the direct classification of the CAP cycles from the EEG signal (using directly, the signal or features extracted from the signal as the classifier input) and was presented by Karimzadeh et al. [17], studying multiple entropy features and classifiers. It was verified that the most relevant features were the Kolmogorov, Shannon and sample entropies while the classification based on the k-nearest neighbors attained the best results. The second approach is the most commonly found in the state of the art approach and is based on two steps. The first involves the detection of the CAP phases and the second applies the CAP scoring rules to the identified phases to determine the CAP cycles. The second approach provides more information (CAP cycles and CAP phases) for the clinical diagnosis. Thus, it was used in this work.

A common practice for CAP phase detection consists of the analysis of the characteristic EEG frequency bands to extract features such as band descriptors [18,19]; Hjorth activity [20]; discrete wavelet transform to estimate an activity index [21]; similarity analysis [13]; tunable thresholds applied to the EEG signal [22]; differential variance [23]; Teager energy operator [24]; Lempel–Ziv complexity [25]; Shannon entropy [26]; empirical mode decomposition [26]; sample entropy [25]; Tsallis entropy [25]; fractal dimension [25]; and log-energy entropy [16].

The features produced are then fed to a classifier for the CAP phase detection, with the most commonly used being tunable threshold, support vector machine, feedforward neural network and linear discriminant analysis. However, there is a gap in state of the art systems regarding the efficient application of a deep learning method. A methodology was proposed by Mostafa et al. [27], directly classifying the EEG signal by a deep stacked autoencoder. Therefore, there is no need for handcraft features and the classifiers learn the relevant patterns directly from the data. Nevertheless, the results reported are too inaccurate for clinical analysis. Hence, the approach followed in this work considers deep learning models for automatic CAP analysis that can be employed for medical examination.

Multiple devices have been proposed for sleep quality estimation, commonly using duration metrics (such as total sleep time) as the reference for the assessment. From the commercially available HMDs, the actigraphs have the most popularity among consumers, mostly due to the easy to use, self-assembly approach that is employed in these devices [11]. However, it was reported that the validity of these tools for sleep quality estimation still requires a systematic examination [28]. It was also established that duration, intensity and continuity measures have a small relationship with subjective ratings of prior-night sleep quality [29]. Therefore, the stability measures (such as metrics related to CAP) could be more significant for medical diagnoses [30].

The line of thought that led to the development of this work was: assessment of sleep quality deficits is considered to be a priority for the future of health care [11]; most methods proposed which are the state of the art for sleep quality analysis are based on duration metrics [11]; however, stability metrics could be more significant for clinical diagnosis [30]; CAP is considered to be one of the most relevant sleep stability indicators [12]; implementation of CAP analysis in a HMD could increase the accessibility of the population to sleep quality examination, allowing the assessment of sleep quality deficits and assisting in the treatment of sleep related disorders [31].

The gaps identified in the state of the art were: medical analysis of the CAP is commonly performed through visual examination of the EEG signal, a process prone to error [13]; although some methods for automated CAP analysis were previously proposed, none of them were implemented in a HMD; most of the proposed methods perform the CAP phases assessment and very few estimate the CAP cycles (highly significant information for sleep quality estimation [5,11]). By taking into consideration the current gaps in the state of the art, the objective of this work was to develop and implement (in a low cost portable HMD) an automatic scoring algorithm for CAP detection (for both CAP phases and cycles). This device is the first that allows the user to self-assemble the sensors and perform the CAP analysis at home. The results (and the EEG signal) can later be examined by a physician for further validation.

The paper is organized as follows: the description of the materials and methods is presented in Section 2; Section 3 presents the performance of the algorithms developed; a discussion of the results is performed in Section 4; the HMD developed is described in Section 5; the paper is concluded in the final section.

## 2. Materials and Methods

The second approach for CAP analysis (first detect the CAP phases, and then apply the CAP scoring rules to determine the CAP cycles), identified in the introduction, was followed in this work, since it can provide more information regarding sleep analysis (CAP phases and CAP cycles). Therefore, an algorithm to predict both the CAP phases and cycles was developed using a featureless method. This method was chosen to create a model because it does not require a feature creation step with a priori knowledge provided by the researchers. Therefore, the model learns the relevant patterns directly from the data [32].

**Figure 2:**
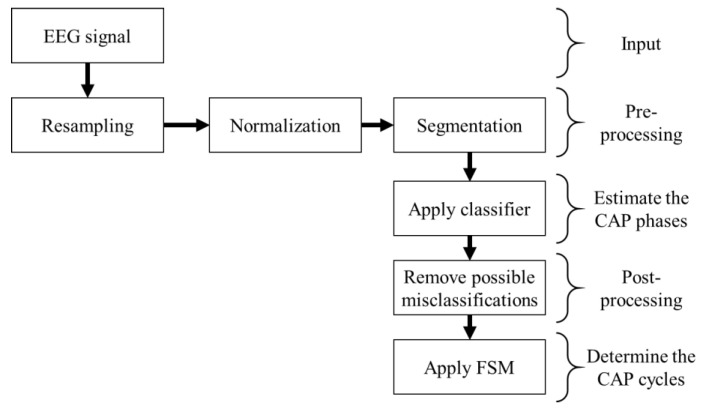
Block diagram representation of the algorithm developed.

The pre-processed signal from an EEG monopolar derivation was fed to a classifier, assessing if each one-second epoch was either an A or a B phase. The classified epochs were then fed to a finite state machine (FSM) to determine the CAP cycles, by applying the CAP scoring rules [5], classifying each epoch as either CAP or not-CAP. The block diagram of the algorithm is presented in Figure 2.

Three machine learning based classifiers were tested, using recordings from a database, for the CAP phase prediction, specifically, the long short-term memory (LSTM), the gated recurrent unit (GRU) and the one-dimension convolutional neural network (1D-CNN). The first two are variations of the recurrent neural networks (RNN) that allow the detection of the temporal correlation components that occur in the physiological signals [33]. The last classifier employs convolution kernels to perform a transformation of the inputs, allowing one to detect the most relevant patterns of the physiological signals and reducing the redundancy through pooling operations [34]. Therefore, two different approaches were analyzed, having the capability to capture complex patterns and create predictions by directly analyzing the signal, without the need for handcrafted features that could be significantly computational demanding and unviable for an HMD with a low price and complexity.

The model that achieved the best performance was, then, implemented in a portable HMD, composed of a sensing unit and a processing unit that wirelessly communicates by Bluetooth, allowing it to perform a reliable prediction of the sleep quality in the subjects’ home.

### 2.1. Database

Full-night recordings from nineteen subjects (ten males and nine females), fifteen free of any neurological disorder (with good and bad sleep quality examples) and four diagnosed with sleep disorder breathing (all with poor sleep quality and the disorder was selected since it is one of the most common sleep related disorders and it has a characteristic CAP pattern [35]), that are available in the CAP Sleep Database [5,36], were employed in this work to develop the algorithms.

This database, from PhysioNet, is the only one that is publicly available and has annotations (created by a team of trained neurologists) for both the micro and macrostructure of sleep. It is also the database commonly used by researchers for CAP analysis. The subject’s average age was 40.57 years old, ranging from 23 to 78 years old to ensure a proper representation of the population. The mean duration of the recordings was 454.07 min, ranging from 220 to 574 min. The scoring rules defined by Terzano et al. [5] were applied to create the CAP cycles annotations from the A phase annotations that are available in the database.

Several signals were recorded during the PSG. However, in this work, only the EEG signals (recorded from one EEG monopolar derivation, either C4-A1 or C3-A2, according to the 10–20 international system [37]), were used, since CAP is a characteristic pattern from these signals. They were recorded with different devices, and consequently, the sampling frequency ranged from 100 to 512 Hz.

### 2.2. Pre-Processing

Since the signals were recorded with different sampling frequencies, a uniform database was created by resampling all of EEG signals at 100 Hz (lowest sampling frequency of the recordings that is also the sampling frequency of the sensor employed in the HMD) for fair comparison and to reduce any frequency dependent components from the results. This approach (resampling at a lower resolution and re-quantizing all signals if needed) also allows one to develop a device-independent estimation of the EEG signal, as indicated by Chapotot et al. [38].

The resampling process was performed by decimation [39], considering a constant factor of reduction of the sample rate *r*. A lowpass filter was employed to downsample the signal and guard against aliasing. However, the artifacts related to movements were kept since they are usually related to the occurrence of a microstructure event [40]. Afterwards, the resampling process selects every *r*th point from the filtered signal. The standard filter was employed, specifically, an order 8 Chebyshev type I filter, with a passband ripple of 0.05 dB and a normalized cutoff frequency of 0.8/*r* [41].

Subsequently, a normalization was performed (subtract the average and divide by the standard deviation) to improve the performance of the classifier. The signal was segmented into one second epochs (without overlapping) to match the labels provided for the microstructure.

### 2.3. CAP Phase Detection

Three machine learning based classifiers (LSTM, GRU and CNN) were tested for the A phase detection by directly analyzing the pre-processed signal. Theoretically, the CAP phases have a temporal dependency [5]. Therefore, two variants of RNN, LSTM and GRU were chosen, since they allow the detection of both short and long-term correlations in time-series [42]. Multiple classifiers, based on feature analysis (including supervised and unsupervised models), for the CAP phase detection, were previously tested by Mendonça et al. [16], concluding that the models based on neural networks attained the best performance. However, the CNN, one of the common feature extraction techniques used in deep learning [43], was not previously tested. Therefore, the 1D-CNN was selected since it is one of the best networks for automatic feature extraction [43] that was, at this time, not yet used for the CAP phase detection. For all classifiers, each epoch was categorized as either A phase or non-A phase (considered to be B phase epoch). Consequently, binary classification was used.

The LSTM memory cell is controlled by three gates: the input (*I*) and output (*O*) gates that, respectively, regulate the flow of activations into the memory cell and from the memory cell to the remaining network; and the forget (*F*) gate that adaptively resets the cell’s memory [42]. At time step *t* the input signal, *x_t_*, is hosted in the cell candidate, *P*, and can update the cell state, *c_t_*, by [42]

(1) $$c_{t}=F_{t}c_{t-1}+I_{t}P_{t}$$

Each cell candidate and gate have unique biases (*B*) and weights (for the recurrence, *R*, and inputs, *W*) that are tuned during the training process. The input and output gates perform a scaling of the internal state through the operations [42]

(2) $$I_{t}=\sigma \left(W_{I}x_{t}+R_{I}h_{t-1}+B_{I}\right)$$

(3) $$O_{t}=\sigma \left(W_{O}x_{t}+R_{O}h_{t-1}+B_{O}\right)$$

where *σ* defines the sigmoid function [44], and the hidden state, *h*, is set by

(4) $$h_{t}=O_{t}tanh\left(c_{t}\right)$$

where *tanh* defines the hyperbolic tangent function [44]. Similarly, the forget gate and cell candidate perform the operations [42]

(5) $$F_{t}=\sigma \left(W_{F}x_{t}+R_{F}h_{t-1}+B_{F}\right)$$

(6) $$P_{t}=\sigma \left(W_{P}x_{t}+R_{P}h_{t-1}+B_{P}\right).$$

The GRU only employs two gated units, reset (*S*) and update (*U*) gates, to modulate the flow of information inside the unit. Therefore, a simpler structure is used and the network can manipulate the number of previous output activations that is preserved through the learned reset gates. A different approach is followed by a LSTM unit that calculates the current cell state by using all of the information flowing from previous outputs without a specific selection [45].

The reset gate (determines if the previous hidden state should be ignored, dropping the information that is considered to be irrelevant later in the future, and therefore, providing a more compact representation) and update gate (controls the amount of information that will be carried over to the current hidden state, from the previous hidden state) also perform a scaling operation, and the activation vectors are given by [46]

(7) $$S_{t}=\sigma \left(W_{S}x_{t}+R_{S}h_{t-1}+B_{S}\right)$$

(8) $$U_{t}=\sigma \left(W_{U}x_{t}+R_{U}h_{t-1}+B_{U}\right)$$

The candidate activation, *M*, is composed of the information learne and the network inputs. It is given by [46]

(9) $$M_{t}=tanh\left(W_{M}x_{t}+R_{M}S_{t}h_{t-1}+B_{M}\right)$$

where [46]

(10) $$h_{t}=\left(1-U_{t}\right)h_{t-1}+U_{t}M_{t}$$

For the 1D-CNN the convolution operation, performed on the convolution layers, is represented by [47]

(11) $$c_{d}=\varphi \left(K_{d}⊛X+B_{d}\right)$$

where *X* are the inputs of the epoch, *φ* is the activation function (in this work the rectified linear unit (RELU) [44] and the scaled exponential linear unit (SELU) [48] were considered) and 1 ≤ *d* ≤ *nK_d_*, bearing in mind that *nK_d_* is the number of convolution kernels, *K* is the kernel, *n* is the dimension of the input and ⊛ is the *𝑛* dimensional convolution operation. The activations of the previous layer are commonly normalized in the batch normalization layer to maintain the mean activation close to zero with an approximately unitary standard deviation [47].

A pooling operation is performed after the convolution and normalization layers to reduce the dimensionality of the data and it commonly implements either the maximum (MaxP) or the average (AveP) of the inputs with a chosen pooling size [47].

Fully connected (dense) layers were employed at the end of each classifier to improve the learning capability of the nonlinear parameter and perform the classification. The layer output is given by [47]

(12) Y=φ(W×X+B)

where *W* and *B* are the weights matrix and the bias vector, respectively, and *φ* is the activation function.

### 2.4. Post-Processing

A post-processing procedure was employed to reduce the CAP phase misclassifications since it was verified by Mendonça et al. [16] that it can improve the performance of the model. An epoch was considered as a misclassification if it denoted an isolated CAP phase; that is, a B phase (with a two second duration) surrounded by two valid A phases or an A phase (that lasts two seconds) bounded by two valid B phases. Therefore, the misclassified epochs were converted into the opposite phases (a B into an A phase and an A into a B phase) since binary classification was used [16].

### 2.5. CAP Cycles

**Figure 3:**
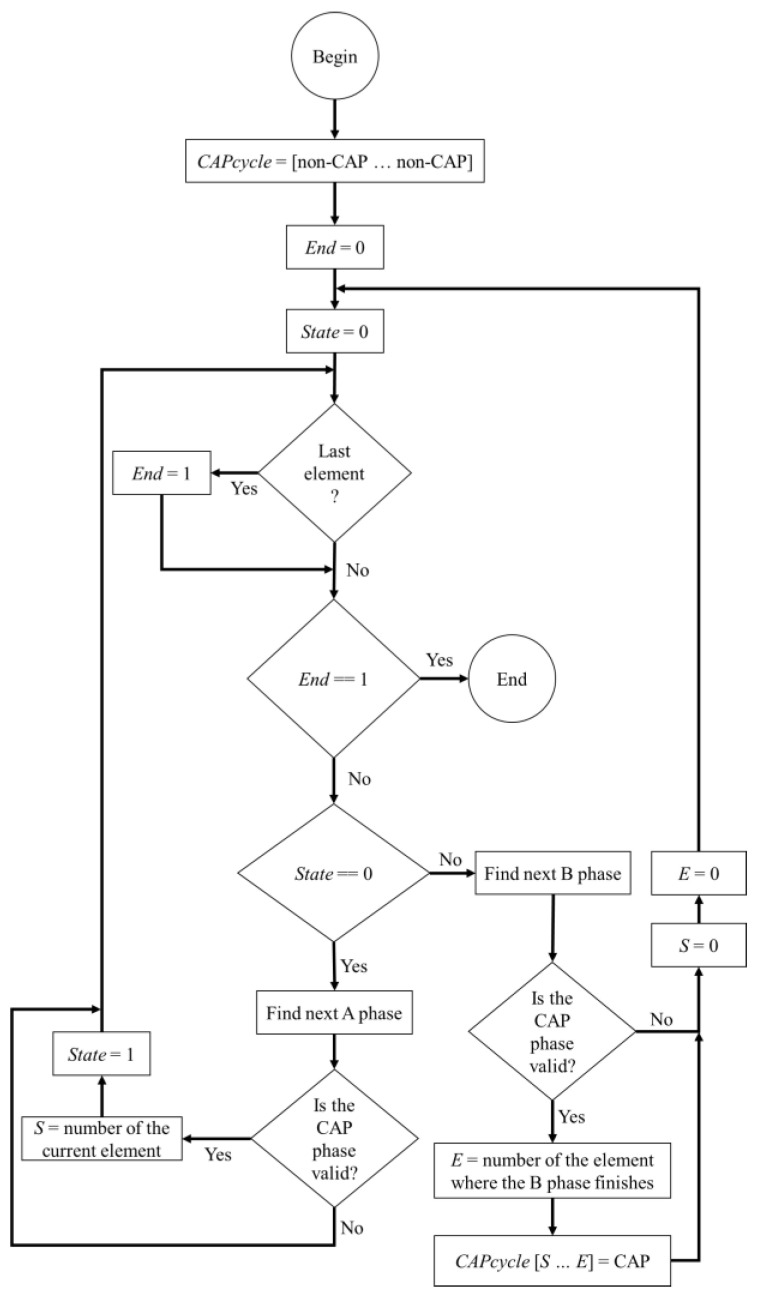
Flowchart of the implemented finite state machine (FSM) algorithm.

The protocol followed to classify the CAP cycles was defined by Terzano et al. [5]. A FSM was implemented to classify the CAP cycles, since the protocol is a rule based method and each rule can be applied by a group of states at any given time. The algorithm flowchart is presented in Figure 3 and the goal is to correctly determine the elements of a vector (named CAPcycles) whose length is equal to the number of epochs that were classified.

In the beginning, all the elements of CAPcycles are marked as non-CAP, referring to an epoch that is not related to a CAP cycle, and iteratively, the FSM runs through all the epochs. If all the conditions for a valid CAP cycle are met, then all the corresponding epochs in CAPcycles, change from non-CAP to CAP (indicating that the epoch belongs to a valid CAP cycle). In the end, the CAPcycles vector comprises the indications regarding the presence of a valid CAP cycle for each epoch.

### 2.6. Performance Evaluation

The standard metrics [49] were analyzed to assess the performances of the models developed for the detection of both the CAP phases and cycles. These metrics were the accuracy (*Acc*), sensitivity (*Sen*), specificity (*Spe*), positive predictive value (*PPV*) and negative predictive value (*NPV*). They were based on the assessment of the true positives (*TP*), true negatives (*TN*), false positives (*FP*) and false negatives (*FN*), and are given by [49]

(13) $$Acc=\frac{TP+TN}{TP+TN+FP+FN}$$

(14) $$Sen=\frac{TP}{TP+FN}$$

(15) $$Spe=\frac{TN}{TN+FP}$$

(16) $$PPV=\frac{TP}{TP+FP}$$

(17) $$NPV=\frac{TN}{TN+FN}$$

The diagnostic odds ratio (*DOR*), a metric that does not depend on the prevalence of the disease, was also used to evaluate the predictions of the models and is defined by [50]

(18) DOR=TPFNFPTN

The diagnostic ability of the model was assessed by the area under the receiver operating characteristic curve (AUC) and it describes the probability of the classifier to rank an arbitrarily chosen positive instance higher than an arbitrarily chosen negative instance [51]. The average value and the 95% confidence interval (CI) were presented for each metric to provide information about the statistical significance of the results [52].

The classifier hyperparameters (including the number of neurons in the hidden layers and the number of layers) were selected by a grid search approach (we performed a simulation for each combination of the hyperparameters to evaluate all possibilities). This optimization was done by choosing the configuration that provided the highest AUC of multiple simulations with cross-validation (considered to be a suitable metric since a general measure of predictiveness was desired [51]).

A leave one out method was used for training and testing of the classifiers because it is the more suitable methods for models with small numbers of samples (common in bioinformatics) since it provides less biased results [53]. Eighteen subjects were chosen for training and one subject was selected for testing. Each training round and test was repeated fifty times to achieve statistically significant results. This process was performed nineteen times to have a test result for all nineteen subjects. The average value of the nineteen iterations was selected as the value for the simulation. This way, subject independence was guaranteed by only using the signal from each subject either in the testing set or in the training set.

## 3. Results

The grid search method employed first chooses a type of layer. After that, it determines the hyperparameters that maximize the AUC for the layer. For the LSTM, GRU and fully connected layers the number of neurons in the layer was varied from 50 to 500 in steps of 50, for the first hidden layer, and from 10 to 100, in steps of 10, for the further hidden layers. For the 1D-CNN the number of filters used in the convolution layer was chosen to be a power of two (for optimization), ranging from eight to 256, while the filter length was varied between one and 10 with a stride of one. The output layer of all the networks was composed of a fully connected layer that applied the Softmax function [47] for classification. A batch normalization layer was used after the convolution layer and an activation function was employed, followed by either MaxP or AveP. The analyzed activation functions were: *σ*; tanh; SELU; RELU.

After the first layer was selected and optimized, a new layer was introduced, and the process repeated. If the new network provided a higher AUC (after the hyperparameters were all tested in the two layers), then this model was selected as the best and the process was repeated by adding another layer. This algorithm iterates until the AUC no longer increases.

Five optimization algorithms commonly employed for deep learning were tested on this work; specifically: stochastic gradient descent (SGD) with and without momentum [47]; RMSprop [54]; adaptive gradient (AdaGrad) [55]; ADADELTA [54]; Adam [56]. It was verified that Adam outperformed the other algorithms for all the networks. However, a similar performance was attained using the RMSprop in the recurrent networks.

The models were developed in Python, using the Keras library, and the structures of the best networks of each type analyzed (LSTM, GRU and 1D-CNN) are presented in Table 1. The implementation was based on a sequential model and each new layer was stacked on top of the previous using the “add” function. In the end, the “compile” function was used to create the network that was trained using the “fit” function. The development of the models took, approximately, three months. The 1D-CNN was the model that took longer to be developed, while the GRU was the model that was developed faster (due to the lower computational time to perform the simulations). It was verified that the best network configuration for both LSTM and GRU was attained using four layers, while eight layers were used for the 1D-CNN. The performance metrics of the three networks for the recognition of CAP phases are presented in Table 2; the performance of the FSM, fed with the post-processed output of the networks, for the detection of the CAP cycles, is presented in Table 3.

It was verified that LSTM achieved the best results (highest AUC, indicating balanced results, and highest *DOR*, suggesting better discriminatory performance) for both CAP phases and cycle detection, while 1D-CNN presented the lowest variation in the CI (difference between the lowest and the highest bounds of the CI) and the lowest AUC for the CAP phase estimation. The highest variation of the CI and difference between *Sen* and *Spe* was attained by GRU, suggesting unbalanced results that are not suitable for clinical diagnosis. The results also advocate that both the CAP phases and cycles have a temporal dependency, agreeing with Tezano et al. [5]. However, the features extracted from the 1D-CNN also allowed us to achieve significant results, proposing that relevant patterns are present in the EEG signal, as suggested by Mariani et al. [8].

## 4. Discussion

Multiple approaches for the CAP phase detection were identified in a review [16], with a global *Acc*, *Sen* and *Spe* (means ± standard deviations) of, respectively, 76% ± 6%, 72% ± 8% and 78% ± 7%. Therefore, the model developed based on the LSTM achieved a higher *Acc* and *Sen* with a slightly lower *Spe*, while the model based on the GRU achieved both a higher *Acc* and *Spe* but a lower *Sen*.

Most of the work found with the state of the art methods removes the REM sleep periods from the analysis (since CAP is only defined in the NREM sleep periods), reducing the misclassifications, and consequently, increasing the *Acc*. However, to achieve a fully automated CAP scoring method a second classifier would have to be implemented to detect the REM periods, and taking into consideration that the agreement among expert scoring the sleep stages from the same EEG signal is lower than 90% [11], it is expected that this approach would introduce more errors. Another relevant factor is the characteristic unbalanced data in healthy subjects, having significantly more B phases than A phases, indicating that an increase in *Spe* has a larger influence in the *Acc* than an increase in *Sen*.

It was verified that the majority of the misclassifications occur in the CAP events boundary, agreeing with the conclusions reported by Largo et al. [15] about the importance of accurately measuring these boundaries. This issue was mitigated by the post-processing procedure employed that provided, on average, an increase of 2% in the *Acc*.

Regarding the CAP cycles’ detection, the average *Acc* identified in a review [16] was 75% ± 5% considering the feature-based approach. The only featureless method found was presented by Mostafa et al. [27] with an average accuracy of 62%. Therefore, the method we developed based on an LSTM achieved, on average, a better performance than both feature and featureless-based methods reported to be the state of the art. It is also relevant that this performance is within the expected mutual agreement range (69% to 77.5%) between two clinicians analyzing the same signals [14].

However, Largo et al. [15] have verified that the global average of the pairwise inter-scorer agreement from seven specialists performing visual scoring was 69.9%, suggesting that the agreement gets closer to the lower bound (69%) as the number of experts that are performing the scoring increases. Hence, the method we developed is, at least, as good as a group of specialists analyzing the same signals and the implementation in an HMD could possibly be used as a diagnostic tool, potentiating the detection and treatment of sleep quality deficits.

## 5. Development of the Home Monitoring Device

**Figure 4:**
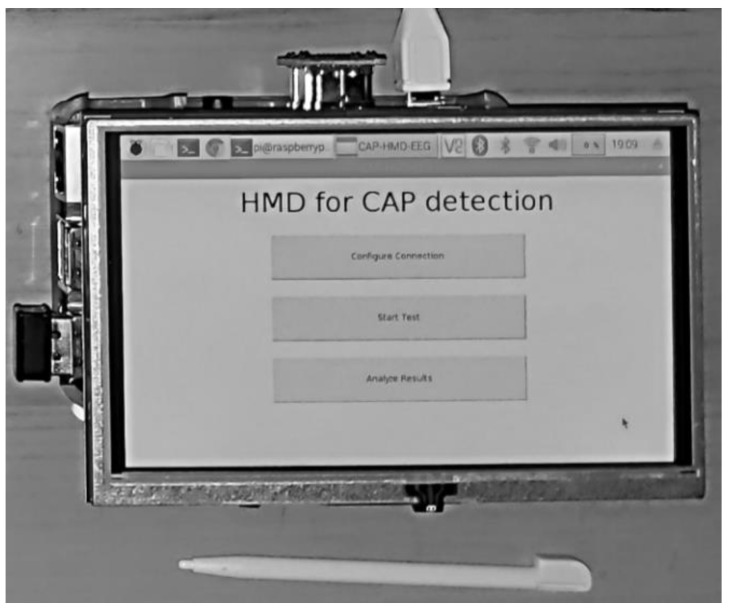
Graphical user interface of the application developed.

The objective was to develop a low cost minimally invasive HMD for automatic CAP detection, using the model that achieved the best performance (LSTM and FSM) to perform the predictions in the processing unit, analyzing the signals that were measured in the sensing unit. The two units wirelessly communicate by Bluetooth, allowing the sensing unit to be small (since minimum processing is needed) and easily self-assembled. The processing unit has a touchscreen that displays the graphical user interface (GUI), presented in Figure 4, where the user can configure the connection, start/stop the exam and analyze the results.

**Figure 5:**
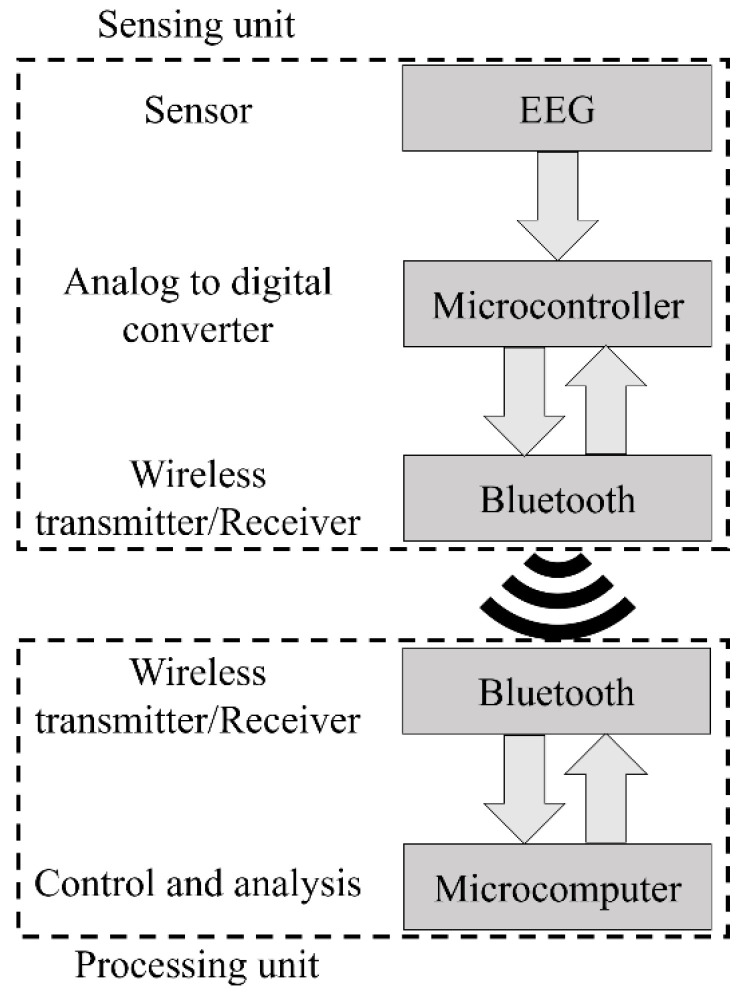
Architecture of the home monitoring device we developed.

**Figure 6:**
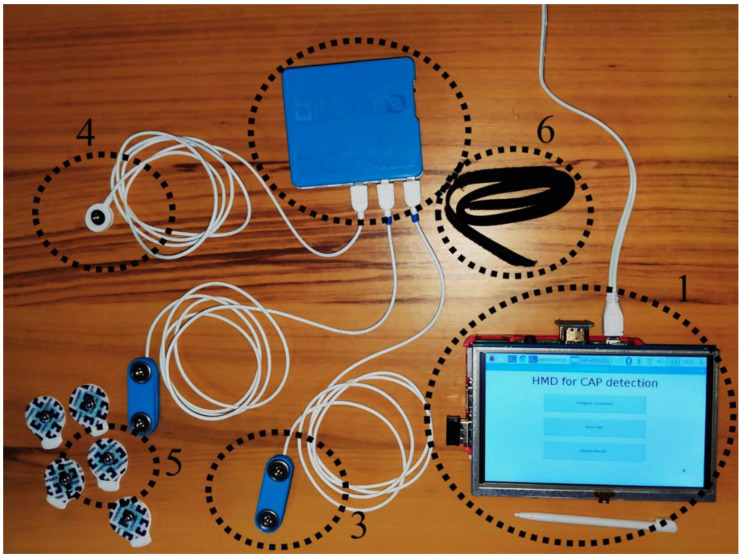
Implemented hardware. (**1**) Processing unit, (**2**) sensing unit, (**3**) electroencephalography sensor, (**4**) ground cable, (**5**) electrode and (**6**) headband.

The system architecture is presented in Figure 5, while Figure 6 presents the implemented hardware. The BITalino Core BT [57] was employed in the sensing unit and it is composed of an ATmega328P microcontroller, a communication block (for Bluetooth communications) and a power management block, all feed by a 3.7 V lithium ion battery, having an average 50 mAh load current (the unit lasts seventeen hours in real-time acquisition over Bluetooth [57]). The device sampling rate is configurable and can be specified by the user in the GUI (the default value is 100 Hz). The number of bits of the analog to digital conversion (ADC), either ten or six bits, is dependent upon the used analog port. For this work, only the ten bit ports were chosen.

**Figure 7:**
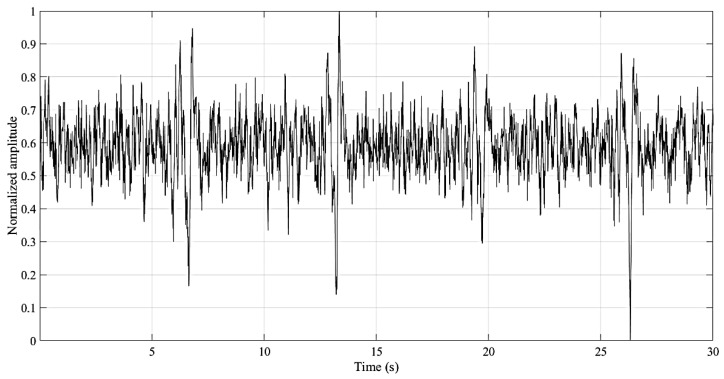
Example of the signal recoded by the home monitoring device (HMD).

The ECG sensors detect the electrical potentials (through the electrode) in the scalp with respect to a ground reference (ground cable), and it was verified, when comparing with an established gold standard device (BioPac MP35 Student Lab Pro), that the average measurement root mean squared error was 0.013 ± 0.005 [58]. This small error advocates the viability of the sensor for clinical diagnosis. An example of the recorded signal is presented in Figure 7. The input noise, common mode rejection ratio and the input impedance of the amplifier employed on the EEG sensor are 50 nV/√Hz, 100 dB and >100 GOhm, respectively.

The processing unit is composed of a single-board computer (Raspberry Pi 3 B+ with 1.4 GHz, 64-bit, ARM quad-core processor), fed by the DC power supply, and a touch screen that displays the GUI where the user interacts with the developed Python application that automatically connects with the sensing unit once it is opened. The applications allow the user to change the ADCs that will be used and specify a new bit rate for the Bluetooth communication (the default value is 19,200 bits/s). However, for the typical examination, the user just has to follow these steps: (1) place the EEG sensor in the C4 or C3 positions (the other EEG sensor, connected to the second ADC, can be placed in another location of interest for clinical diagnosis, such as Fp1 or Fp2, but it will not be considered for the device CAP detection), according to the 10–20 international system; (2) tighten the headband around the sensor to decrease the measurement noise; (3) place the ground electrode in the mastoid region (A1 if the EEG sensor was placed on C4, or A2 otherwise); (4) turn on the sensing unit; (5) turn on the processing unit and wait until the “HMD for CAP detection” application is open; press in the “Start Test” button (a new window will appear with the “Stop Test” button); (6) when the test is finished press the “Stop Test” button (all the information that is received in the sensing unit is stored in a text file with a timestamp on the 32 GB secure digital memory card); (7) press the “Analyze Results” button (the application opens the text file, stores the signal in a variable and uses the developed algorithm for CAP detection) and wait until the “Finished Analyzing the Results” message is displayed (the predicated CAP phases and cycles are stored, with a timestamp, in a text file).

**Figure 8:**
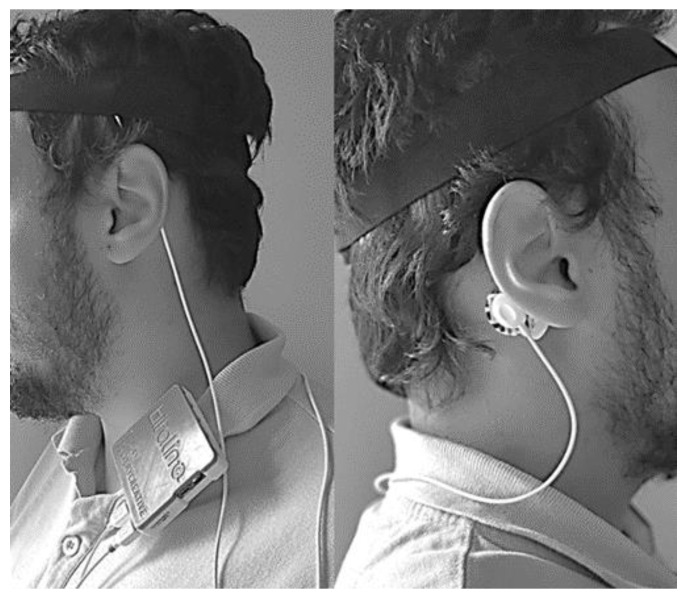
Example of how to assemble the sensing unit on a subject.

An example of how to assemble the sensing unit in a subject is presented in Figure 8 (fixing the sensing unit, by using the s-shaped belt clip, on the shirt). At the end of the test the user can either analyze the results file or provide the device to an expert to retrieve the file and make the analysis of the results. Since the device is intended to be self-assembled by the user, there is always the possible issue of poor contact between the electrode and the scalp due to bad assembly. If the signal recorded was close to zero for more than sixty consecutive epochs, the respective sixty epochs are marked as erroneous in the CAP scoring file and the device assumes that an electrode has popped off. A new one minute cycle begins, and, if the recorded signal still has no variations (remains close to zero), the sixty epochs will again be marked as errors. This process is repeated until the signals presents variations or until the end of the recorded data (for the case were the electrode is poorly placed and the recording is not adequate).

The cost of the processing unit was 60 €, while the sensing unit’s cost was 220 €. However, the total cost of a commercial product could be significantly reduced by developing the hardware of the sensing unit with only the EEG sensor connected to a communications module. Nevertheless, a new sensing unit will require a validation by performing a parallel recording with the PSG; meanwhile, the module has already been validated. A brief summary of other commercial, off-the-shelf EEG sensors is presented in Table 4 (indicated prices were attained from the official company store or from contacting authorized sales consultants; when the device has more than one version, the one with less recording channels is presented, since it is more suitable for the work). However, they are all either not validated or are significantly more expensive than the sensor used. The significance of the HMD developed is augment by the fact that no other device, capable of monitoring CAP events at the patient’s home, was found in the literature [11].

## 6. Conclusions

An automatic diagnostic system, based on the analysis of an EEG monopolar derivation signal for the detection of the CAP phases and cycles, was developed. Three featureless-based classifiers were tested and the one that achieved the best performance (highest AUC) was implemented in the HMD to produce an automatic CAP scorer.

It was verified that CAP has a strong temporal dependency, as indicated by Tezano et al. [5], that allowed the LSTM based classifier to achieve an average performance that is in the higher bound of the experts’ agreement range indicated by Rosa et al. [14] and significantly higher than the inter-scorer agreement of multiple experts reported by Largo et al. [15]. It is also a relevant fact that the algorithm does not need to remove the REM periods (common practice for CAP scoring), making the implementation more suitable for clinical analysis.

It is intended that the developed HMD will be used as a diagnostic tool and lead to the diagnosis and treatment of sleep quality deficits. However, it was also verified that most misclassifications happen in the CAP phase boundaries, possibly suggesting that bigger windows (for instance, epochs that last one minute and apply a tuned threshold to determine if the epochs are either CAP or non-CAP, as employed by Mendonça et al. [59]) could possibly improve the performance.

On a utility test it was verified that the HMD was effortlessly self-assembled, easy to operate and properly recorded the EEG signal. The next step in the research is the validation of a new version of the device, developed according to the IEC 80601-2-26:2019 standard [60], performing recordings with the PSG in parallel with the device; and determining the viability of a system that directly detects the CAP cycles. We also intended to introduce an alarm in the device that alerts the user when the quality of the EEG signal is inadequate (possibly due to poor contact between the electrode and the scalp), requiring the user to reassemble the sensors.

## Figures and Tables

**Table 1:** Structure of the classifiers that provided the highest areas under the receiver operating characteristic curves (AUCs).

Type of Network		
LSTM	GRU	1D-CNN
Input (100 neurons) LSTM (400 cells) Fully connected (30 neurons) SELU Output Softmax	Input (100 neurons) GRU (200 cells) Fully connected (20 neurons) SELU Output Softmax	Input (100) Convolution (64 filters with length 3) Batch normalization (64 channels) RELU MaxP (pool size = 3) Convolution (128 filters with length 3) Batch normalization (128 channels) RELU Global AveP Output Softmax

**Table 2:** Performance of networks for the CAP phases’ estimation.

Type of Network	Performance Metrics [Mean (95% CI)]						
	*Acc* (%)	*Sen* (%)	*Spe* (%)	*PPV* (%)	*NPV* (%)	*AUC*	*DOR*
LSTM	76.0 (74.3–77.7)	74.6 (71.5–77.6)	76.6 (74.9–78.3)	65.9 (62.8–69.0)	82.1 (80.0–84.1)	0.752 (0.749–0.755)	9.61 (9.02–10.21)
GRU	80.7 (78.5–82.9)	65.5 (62.1–68.9)	83.0 (78.7–87.3)	56.6 (50.8–60.3)	78.5 (73.8–83.2)	0.742 (0.714–0.770)	9.27 (8.41–10.13)
1D-CNN	74.4 (73.4–75.3)	71.2 (70.0–72.3)	74.8 (73.5–76.1)	62.0 (60.3–63.6)	80.0 (78.4–81.2)	0.729 (0.728–0.730)	7.34 (6.86–7.81)

**Table 3:** Performance of the models for the CAP cycles’ estimation.

Type of Network	Performance Metrics [Mean (95% CI)]						
	*Acc* (%)	*Sen* (%)	*Spe* (%)	*PPV* (%)	*NPV* (%)	*AUC*	*DOR*
LSTM and FSM	76.3 (74.2–78.4)	71.4 (68.3–74.5)	84.2 (81.4–87.0)	67.8 (64.5–71.2)	77.7 (74.7–80.7)	0.778 (0.760–0.796)	13.30 (12.61–14.0)
GRU and FSM	71.9 (69.4–74.4)	59.9 (56.0–63.8)	87.8 (84.1–91.5)	55.8 (51.5–60.0)	72.2 (68.2–76.1)	0.738 (0.719–0.757)	10.75 (9.77–11.73)
1D-CNN and FSM	72.5 (71.3–73.7)	65.8 (64.7–66.9)	83.3 (82.0–84.6)	61.9 (60.7–63.2)	74.3 (72.3–75.7)	0.745 (0.741–0.749)	9.60 (9.19–10.0)

**Table 4:** Summary of some EEG sensors available on the market that could be used in the HMD developed.

Device	Company	Price (€)
MindWave Mobile 2	NeuroSky (USA)	91
Insight	Emotiv (USA)	271
Epoc+	Emotiv (USA)	725
BrainStatus	Bittium (Finland)	867
Hybrid Black	Unicorn (Austria)	990
All-in-One EEG kit	OpenBCI (USA)	2248
Quick-8	Cognionics (USA)	3627
Enobio 8	Neuroelectrics (Spain)	3995
B-Alert X10	Advanced brain monitoring (USA)	9092
ActiveTwo	BioSemi (Netherlands)	13,500
